# Authentication of Edible Oil by Real-Time One Class Classification Modeling

**DOI:** 10.3390/foods14071235

**Published:** 2025-04-01

**Authors:** Min Liu, Xueyan Wang, Yong Yang, Fengqin Tu, Li Yu, Fei Ma, Xuefang Wang, Xiaoming Jiang, Xinjing Dou, Peiwu Li, Liangxiao Zhang

**Affiliations:** 1 Key Laboratory of Edible Oil Quality and Safety, State Administration for Market Regulation, Key Laboratory of Biology and Genetic Improvement of Oil Crops, Ministry of Agriculture and Rural Affairs, Quality Inspection and Test Center for Oilseed Products, Ministry of Agriculture and Rural Affairs, Oil Crops Research Institute, Chinese Academy of Agricultural Sciences, Wuhan 430062, China; 2 Wuhan Institute for Food and Cosmetic Control, Wuhan 430040, China; 3 College of Food and Bioengineering, Henan University of Science and Technology, Luoyang 471000, China; 4 Hubei Hongshan Laboratory, Wuhan 430070, China

**Keywords:** edible oil, authentication, chemical marker, Monte Carlo, one-class partial least squares, model population analysis

## Abstract

Adulteration detection or authentication is considered a type of one-class classification (OCC) in chemometrics. An effective OCC model requires representative samples. However, it is challenging to collect representative samples from all over the world. Moreover, it is also very hard to evaluate the representativeness of collected samples. In this study, we blazed a new trail to propose an authentication method to identify adulterated edible oils without building a prediction model beforehand. An authentication method developed by real-time one-class classification modeling, and model population analysis was designed to identify adulterated oils in the market without building a classification model beforehand. The underlying philosophy of the method is that the sum of the absolute centered residual (ACR) of the good model built by only authentic samples is higher than that of the bad model built by authentic and adulterated samples. In detail, a large number of OCC models were built by selecting partial samples out of inspected samples using Monte Carlo sampling. Then, adulterated samples involved in the test of these good models were identified. Taking the inspected samples of avocado oils as an example, as a result, 6 out of 40 avocado oils were identified as adulterated and then validated by chemical markers. The successful identification of avocado oils adulterated with soybean oil, corn oil, or rapeseed oil validated the effectiveness of our method. The proposed method provides a novel idea for oils as well as other high-value food adulteration detection.

## 1. Introduction

Food fraud is an ancient topic and is still a challenging problem that influences consumers’ health and the healthy development of the industry [1,2]. In particular, economically motivated adulteration (EMA) for some high-value foods-such as seafood, milk, honey, and edible oils was reported at high risk, especially oil adulteration [3,4]. In the previous papers, olive oil, camellia oil, avocado oil, and sesame oil, etc., were reported to be the most likely adulterated targets [4,5,6].

Authentication of edible oils is a challenging analytical problem in food chemistry. In recent years, a wide range of detection methods has been developed, employing diverse analytical techniques. For example, methods based on FT-IR [7], 3D-fluorescence [8,9] laser-induced fluorescence (LIF) [10] Hyperspectral Imaging (HSI), Raman, UV-vis [11], NMR [12], GC and GC-MS [13], LC and LC-MS [14], GC-IMS [15], E-nose and E-tough [16], mass spectrometry imaging (MSI) [17], Differential Scanning Calorimetry (DSC) [18], and laser-induced breakdown spectroscopy (LIBS) [19,20] have all been successfully applied. These methods were often integrated with chemometric analysis to establish the oil authentication method. Each analytical technique possesses advantages and special features: the spectroscopy-based method was typically fast, non-destructive, and cost-effective, whereas the chromatography-mass spectrometry-based methods offered detailed insights into the chemical composition and metabolic profiles of edible oils.

Traditionally, adulteration detection methods have relied on constructing a two-class classification model of authentic and adulterated samples [21]. However, since it is much more difficult to determine the adulterant than to detect adulteration, traditional adulteration detection methods can only detect adulterated oil with one or two targeted adulterants [22]. As a result, adulteration detection or authentication is considered a type of one-class classification (OCC) in chemometrics [23,24]. Recently, OCC models were built to identify the authenticity of peanut oil [25], sesame oil [23,26], and olive oil [27]. In our previous work, two authentication models for Changshan camellia oil were developed by data-driven soft independent modeling of class analogy (DD-SIMCA) and one-class partial least squares (OCPLS) to differentiate Changshan from non-Changshan camellia oil [28]. This means that an effective OCC model requires representative samples. However, it is challenging to collect representative samples from all over the world. Moreover, it is also very hard to evaluate the representativeness of collected samples. Particularly in practical inspections, the oils were typically blind samples, which rendered the detection of adulteration more challenging.

Avocado (Persea americana Mill), commonly known as alligator pear, is a fruit native to Mexico and Central America [29]. It is rich in nutrients beneficial to human health and is a highly profitable crop in both domestic and international markets [30,31]. Avocado oil, an important product derived from avocado, offers various health benefits, including potential applications in osteoarthritis treatment [32], wound healing [33], anti-diabetic effects [34], and cholesterol reduction coupled with liver protection [31]. Additionally, avocado oil has a special flavor and a high smoke point, making it suitable for cooking methods like sizzling or deep frying, thus serving as a viable option for daily edible oil consumption [35]. Given these properties, avocado oil was considered a new functional edible oil, and its demand has increased significantly due to consumer interest in its health benefits. Given its high market value and associated premium price, avocado oil is especially susceptible to adulteration [5,10]. Consequently, there is a pressing need to develop an effective method for the avocado oil authentication.

In this study, we blazed a new trail to propose an authentication method to identify adulterated edible oils without building a prediction model beforehand. Herein, high-value avocado oils serve as the case study to validate our approach. Authentic samples in inspected samples were used to prosecute the adulterated samples. The underlying philosophy of the method is that the sum of absolute centered residual (ACR) of a good OCC model built by only authentic samples is higher than that of a bad OCC model built by authentic and adulterated samples. In detail, a large number of OCC models were built by selecting partial samples (e.g., 40%, 50%, or 60%) out of inspected samples using Monte Carlo sampling. Probabilistically, the good OCC models were built by only authentic samples. Then, by sorting the sum of ACR values of large number of OCC models, we can find these good OCC models and then identify adulterated samples uninvolved in these good models. Chemical markers of several cheaper edible oils were analyzed and used to confirm the adulteration detection results. The proposed approach not only effectively identifies unknown adulteration during practical inspections but also offers a novel method to address the issue of representativeness in training set samples when constructing the model.

## 2. Materials and Methods

### 2.1. Avocado Oil Samples

A total of 40 avocado oils were collected online sources, and the crude oils’ origins included Mexico, New Zealand, France, Australia, the United States, Spain, and Kenya.

### 2.2. Chemicals and Reagents

The standards of 37 fatty acid methyl esters (FAMEs), α-, β-, γ-, and δ-tocopherol, Δ5-avenasterol, brassicasterol, campesterol, cycloartanol, cycloartenol, 24-methylene cycloartanol, β-sitosterol, stigmasterol, and N-trimethylsilyl-N-methylheptafluoro butyramide (MSHFBA) were purchased from Sigma Chemical Co. (St. Louis, MO, USA). Other reagents and solvents were obtained from Sinopharm Chemical Regent Co. (Shanghai, China).

### 2.3. Chemical Composition

The composition of FAMEs was determined using analytical methods described in a previous study [36]. The tocopherol analysis was performed according to a previous study with minor modifications [37]. The phytosterol analysis was performed according to a previous study [38] with minor modifications. Moreover, adulteration verification was conducted using characteristic markers. The isoflavones were determined using liquid chromatography-mass spectrometry (LC-MS) analysis according to our previous study [39]. Oryzanol in edible oil was determined via RP-HPLC [40].

### 2.4. Statistical Analysis

Data analysis was performed using Matlab R2020a (Mathworks, Natick, MA, USA). The Matlab codes for one-class partial least squares (OCPLS) were obtained from a previous study [41]. The values of the different parameters were expressed as the mean. An analysis of variance (ANOVA) was performed using the software IBM SPSS Statistics (Version 21.0; Armonk, NY, USA) to check the statistical significance using Tukey tests at a confidence level of 95.0%.

### 2.5. OCPLS

OCPLS is a class modeling method based on the framework of PLS. Score distance (SD) measures the distance of an object from the class center within the space defined by the primary OCPLS latent variables (LVs), while ACR reflects the variability of projections along the OCPLS regression coefficient vector. The detailed derivation of the statistics and confidence intervals for OCPLS can be found in the previous studies by Xu et al. [41]. In the OCPLS model, the samples with low ACR values were considered regular points (authentic ones) based on the SD and ACR of the predicted response.

## 3. Results and Discussion

### 3.1. Adulteration Detection Theory

In this study, an authentication method was proposed to identify adulterated edible oils. A real-time model was constructed using authentic oils from the same batch during the practical inspection in the market surveillance, ensuring that its application domain encompasses all samples to be evaluated with the assumption that the oil from the same batch featured similar distribution and characteristics (the origin, age, and storage conditions). Furthermore, another hypothetical situation was that there were only a minority limited adulterated oil samples while the majority of oil samples were authentic during practical inspection in normal market surveillance. The underlying philosophy of the method is that the sum of the ACR of an OCC model built by only authentic samples is higher than that of a bad OCC model built by authentic and adulterated samples [41]. Specifically, in OCC modeling, when only authentic oil samples are used to build the model, its decision boundary shows a highly compact feature, and the ACR prediction value of adulterated samples in the test set will be significantly higher than that of authentic oil samples, making it easy to distinguish adulterated samples from authentic oil samples. However, when adulterated samples are mixed into the training set, the formed decision boundary will have an abnormal expansion phenomenon (as the masking effect), and the predicted ACR value of adulterated samples in the test set will converge with that of authentic oil samples. The ACR prediction value of the overall test set shows a systematic reduction, which leads to false negatives where some adulterated samples in the test set are misjudged as authentic oil.

In detail, a large number of OCC models were built by selecting partial samples (e.g., 40%, 50%, or 60%) out of inspected samples using Monte Carlo sampling [28]. Probabilistically, the good OCC models built by only authentic samples exit (as shown in Equation (1) and (2), the probability of the good models ($P_{n}^{x}$), and the theoretical number of the good models (N) could be calculated according to parameters including the total models (T), the adulterated samples (m), the total samples (n), the training set (x), and the sampling ratio (k)). Then, by sorting the sum of ACR values of a large number of OCC models, we can find these good OCC models and then identify authentic samples involved in these good models. Surely, after identifying authentic samples, the adulterated samples were also identified. Our method consists of three steps:

(1) $$N=T\times P_{n}^{x}=T\times \frac{C_{n-m}^{x}}{C_{n}^{x}}=T\times \frac{\frac{\left(n\;-\;m\right)!}{x!\left(n\;-\;m\;-\;x\right)!}}{\frac{n!}{x!\left(n\;-\;x\right)!}}=T\times \frac{\left(n\;-\;m\right)!\left(n\;-\;x\right)!}{\left(n\;-\;m\;-\;x\right)!n!}$$

(2) x=n×k

The first step is to build a large number of OCC models using Monte Carlo sampling. The dataset was randomly divided into a training set and a validation set using a sampling ratio (k) that varied between 40% and 80%. Subsequently, OCC models were constructed using the training set, and the corresponding predicted ACR values for the test set were obtained. This procedure was repeated T times, and the sum absolute centered residual (SACR, see Equation (3), the test set was n − x while the training set was x according to Section 3.1) for the test dataset was recorded for each OCC model.

(3) $$SACR=\sum_{i=1}^{n-x}{ACR}_{i}$$

**Figure 1:**
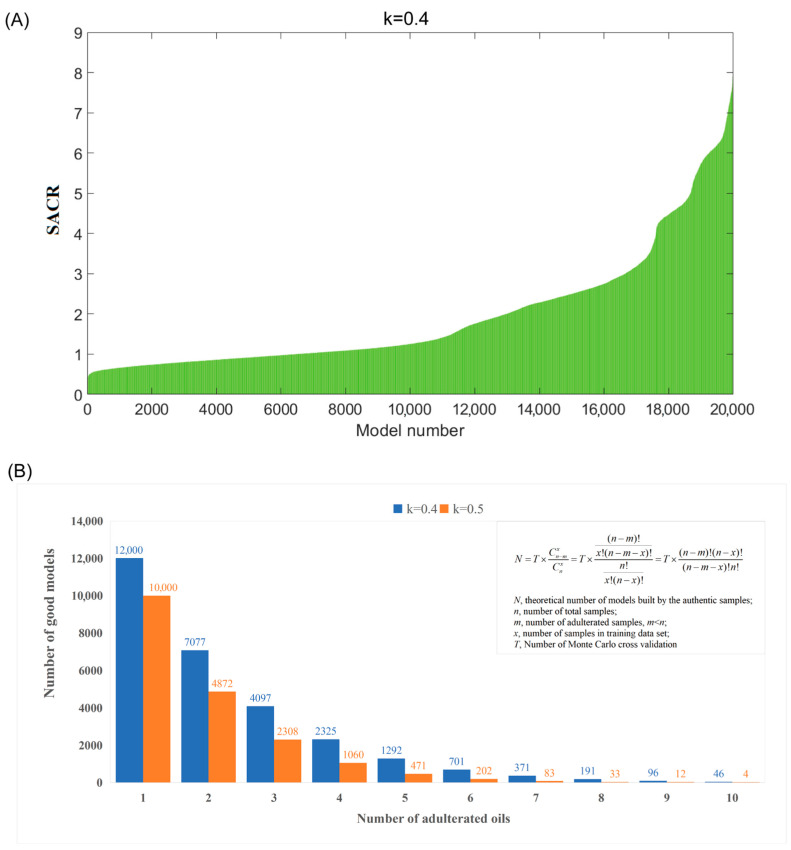
(**A**) The plot of SACR values for the remaining test dataset of 20,000 OCPLS models (k = 0.4); (**B**) the theoretical number of good models calculated based on possible numbers of adulterated oils (from 1 to 10).

The second step involves selecting good OCC models and identifying potential adulterated oils. The models were sorted in ascending order based on their sum ACR values (SACR), and a bar chart was generated displaying SACR values on the y-axis and the corresponding model numbers on the x-axis (Figure 1A). From the bar chart, the models were classified into two groups: those with high SACR values, which were built using only authentic samples (referred to as the “high tiers” in the chart), and those containing adulterated samples (referred to as the “low tiers” in the chart). According to the possible numbers of adulterated oils (m ranging from 1 to 10), the formula shown in Figure 1B was used to compute the theoretical number of good OCC models. Then, a trial-and-error method was employed to determine the number of good models for each possible number of adulterated oils. The likelihood of adulterated oils being uninvolved in the training set of top theoretical good OCC models (which means the adulterated oils were selected as test set samples) will be significantly greater than authentic oils. Specifically, if the probability of an oil sample involved in the test set of good OCC models exceeds 0.8, it could be identified as a potential adulterated oil sample.

The third step is to validate the identified potential adulterated oils by changing the Monte Carlo sampling ratio. To assess the reliability of the results, we proposed altering the sampling ratio k to validate the identification results, as changes in the k value would influence the distribution of effective models within the Monte Carlo model population. When the identified adulterated oils were consistent, then the results could be confirmed.

### 3.2. Adulteration Detection in Inspected Avocado Oil Samples

In order to clarify our method, the inspected samples of avocado oil in our case (n = 40) were taken as an example, and the fatty acid compositions were used as variables to build OCC models (the fatty acid content of authentic avocado oils were presented in Table 1). In detail, the proposed method consists of three steps:

The first step is to build a large number of OCC models. In detail, Monte Carlo sampling was used to randomly select 40% (k = 0.4) of the avocado oils (x = 16), which were then used to construct an OCPLS model for predicting the remaining 60% of the samples (n − x = 24). The sum of ACR values of 24 samples in the validation set was calculated to evaluate this OCPLS model [41]. Monte Carlo sampling was repeated 20,000 times to obtain 20,000 sums of ACR values.

**Figure 2:**
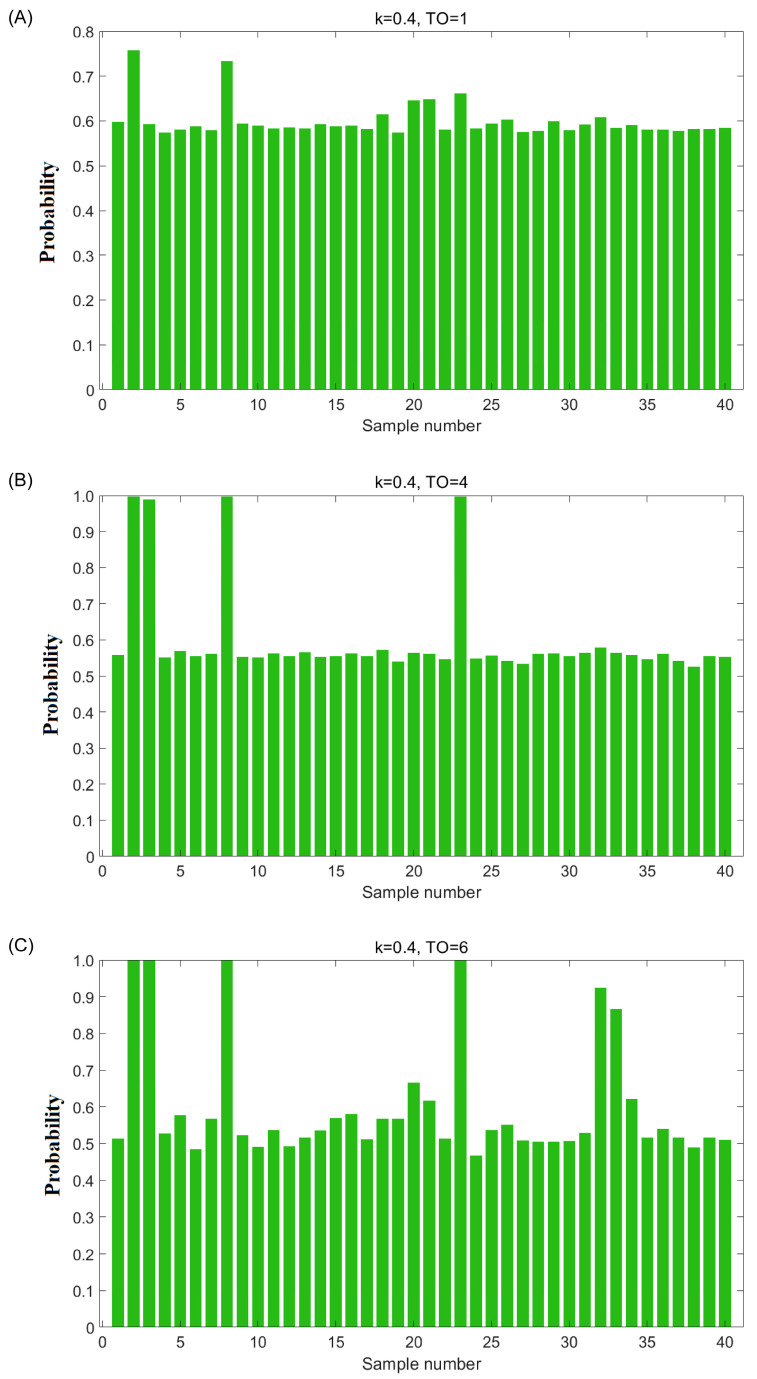
(**A**) The probability of 40 samples of the corresponding theoretically good models (k = 0.4, total number of outliers (TO) = 1); (**B**) the probability of 40 samples of the corresponding theoretically good models (k = 0.4, TO = 4); (**C**) the probability of 40 samples of the corresponding theoretically good models (k = 0.4, TO = 6).

The second step is selecting good OCPLS models and identifying potential adulterated oils. As shown in Figure 1A, 20,000 models were sorted according to the sum of ACR values in ascending order. Generally, the sum of the ACR value of a good OCC model built using authentic samples is higher than that of a bad OCC model built by authentic and adulterated samples. From Figure 1A, it can be seen that the shape of the sum of ACR values resembles a half mountain peak with several tiers, indicating that the adulterated avocado oils disturbed the normal distribution. Therefore, the models in the first tiers on the right-hand side could be regarded as good ones. The key point of this step is to determine the number of good models. Because the total number of samples (n = 40), the Monte Carlo sampling ratio (k = 0.4), and the total number of models (T = 20,000) were known, the theoretical number of good models (N) could be calculated for several possible numbers of adulterated oils (m from 1 to 10), and these values are shown in Figure 1B. Therefore, a trial-and-error method was employed to determine the number of good models. In detail, supposing that the number of adulterated oil is one (m = 1), 12,000 good models exist in theory. The probability of the adulterated oils that were uninvolved in the training set of these good models (in other words, involved in the test set of these good models) will be significantly higher than the authentic ones; specifically, if the probability of the oil sample that was involved in the test set of the assumed-good models is higher than 0.8, then it was considered as a potential adulterated oil sample. The probabilities of each sample uninvolved in these 12,000 good models were calculated and are illustrated in Figure 2A. However, the probabilities of 40 samples were all lower than 0.8, indicating that no sample was identified as adulterated oil. This result is inconsistent with the above assumption; therefore, the assumption does not hold. The same conclusion was obtained when the number of adulterated oils was equal to two or three. When the number of adulterated oils was equal to four (m = 4), the probabilities of each sample involved in the test set of 2325 theoretically good models were calculated, and the results are shown in Figure 2B. In this case, it was found that the probabilities of four samples were higher than 0.8. This number is consistent with the above assumption. When the number of adulterated oils was equal to six (m = 6), the probabilities of each sample involved in the test set of 701 theoretically good models were calculated and are illustrated in Figure 2C. It was found that the probability values of six samples were higher than 0.8, which is consistent with the assumption. Therefore, samples 2, 3, 8, 23, 32, and 33 were identified as potentially adulterated oil.

**Figure 3:**
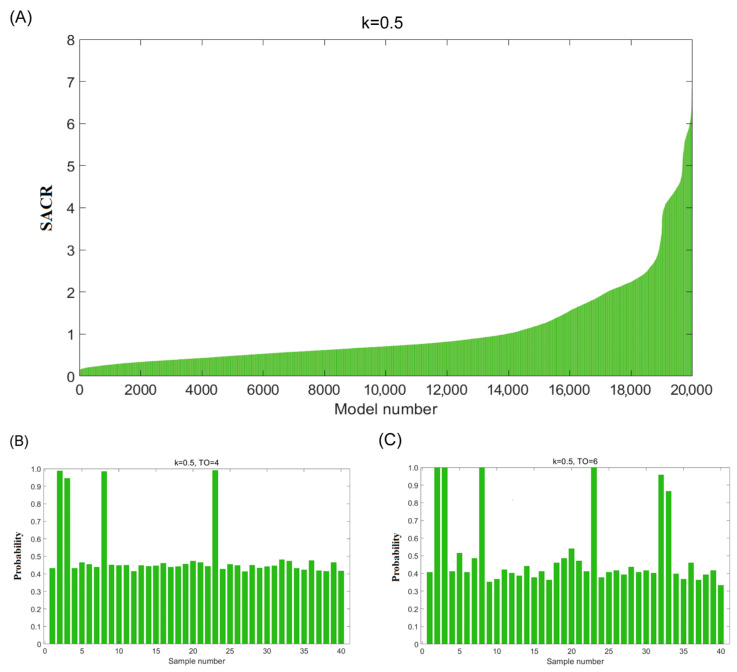
(**A**) The plot of SACR values for the remaining test dataset of 20,000 OCPLS models (k = 0.5); (**B**) the probability of 40 samples of the corresponding theoretically good models (k = 0.5, TO = 4); (**C**) the probability of 40 samples of the corresponding theoretically good models (k = 0.5, TO = 6).

The third step is validating the identified potential adulterated oils by adjusting the Monte Carlo sampling ratio (k = 0.5). Monte Carlo sampling was used to randomly select 50% of the avocado oils (x = 20), which were then used to construct an OCPLS model to predict the remaining 50% of the samples (n − x = 20). A total of 20,000 models were built and sorted according to the sum of ACR values in ascending order (Figure 3A). The number of good models could be calculated using the possible number of adulterated oils (m from 1 to 10), as shown in Figure 1B. With the help of the trial-and-error method, the same conclusions were obtained for numbers of adulterated oils from one to six with a Monte Carlo sampling ratio of 0.4. As illustrated in Figure 3B, when the number of adulterated oil was equal to 4 (m = 4), 4 out of 40 samples from 1060 theoretically good models had high probabilities. Meanwhile, it was found that probabilities of six samples were higher than 0.8, as shown in Figure 3C. The above results validated our determination that samples 2, 3, 8, 23, 32, and 33 were potentially adulterated oil.

### 3.3. Validation by Chemical Markers

Because the avocado oils in this study were purchased from a market, it is necessary to validate the above results by using the chemical markers of several cheap edible oils. According to the content of α-linolenic acid, four samples (2, 3, 8, and 23) possessed higher contents of α-linolenic acid than the other samples and the content of α-linolenic acid in avocado oil in a previous study [29]. Therefore, it was suspected that samples 2, 3, 8, and 23 were adulterated with soybean oil (4.2–11%) and/or rapeseed oil (5–14%). Moreover, our results indicated that the primary tocopherol in avocado oil was α-tocopherol, followed by γ-tocopherol and δ-tocopherol. The contents of γ-tocopherol in six samples were significantly higher than that of the others. The total tocopherol contents of the five samples (2, 3, 8, 32, and 33) were 850.84, 658.81, 1125.56, 843.60, and 936.92 mg/kg, respectively. It was suspected that they might be adulterated with a high-tocopherol oil, such as soybean oil (1411.1 mg/kg) [42]. The contents of α-linolenic acid in samples 2, 3, and 8 were higher than that of others. Hence, it was suspected that soybean oil was added to samples 2, 3, and 8. Furthermore, the range of the total phytosterol contents of avocado oils was from 240.96 to 339.64 mg/100 g [43], while the range of the total phytosterol contents of virgin avocado oils prepared by advanced green technology was from 259 to 360 mg/100 g. In addition, the proportions of campesterol in samples 2, 3, and 8 were 16.3%, 10.8%, and 17.1%, respectively. It was also verified that the content of campesterol increased due to the addition of soybean oil. Moreover, isoflavones are a subgroup of polyphenols, and genistin and daidzin are the two isoflavones found in soybean; thus, these two substances are generally considered to be characteristic markers of soybean oil [39]. However, isoflavones in avocado oil have not been reported. Daidzein and genistein were detected in the samples that we suspected were adulterated with soybean oil. This also verified our conjecture that samples 2, 3, and 8 were adulterated with soybean oil.

Among all samples, only sample 23 contained erucic acid (C22:1) and α-linolenic acid (3.13%), and these were significantly higher than in other authentic avocado oils but are similar to the fatty acid composition of rapeseed oil. It was suspected that sample 23 was mixed with rapeseed oil, and the fact that only sample 23 contained brassicasterol verified this hypothesis.

The total phytosterol contents of samples 32 and 33 were 617.82 mg/100 g and 573.12 mg/100 g, respectively, which were significantly higher than those found in previous studies [36,43]. In addition, the proportion of campesterol in avocado oils was between 3.71% and 6.09%. However, the proportions of campesterol in samples 32 and 33 were 13.9% and 11.4%, respectively, which were significantly higher than the proportion of campesterol reported in previous studies. Therefore, it is speculated that samples 32 and 33 were adulterated with an edible oil with a high total phytosterol content and a high content of campesterol. Among many edible oils, corn oil possesses high contents of tocopherols and phytosterols and has a high proportion of γ-tocopherol and campesterol. It is suspected that samples 32 and 33 were adulterated with corn oil. Three types of oryzanol (cycloartenyl ferulate, campesteryl ferulate, and β-sitosteryl ferulate) were detected in samples 32 and 33, which also verified the conjecture that samples 32 and 33 were adulterated with corn oil. The above validation results indicated that the proposed method could completely identify adulterated samples from inspected samples.

## 4. Conclusions

In this study, an authentication method based on real-time one-class classification modeling and model population analysis was designed to identify adulterated oils in the market without building a classification model beforehand. The underlying philosophy of the method is that the sum of absolute centered residual values of an OCC model built by only authentic samples is higher than that of a bad OCC model built by authentic and adulterated samples. In detail, a large number of OCC models were built by selecting partial samples out of inspected samples using Monte Carlo sampling. Then, by sorting the sum of ACR values of the large number of OCC models, we could find these good OCC models and then identify adulterated samples that were uninvolved in these good models (involved in the test set of the good models). As a result, 6 out of 40 avocado oils were identified as adulterated samples and then validated by chemical markers. The successful identification of avocado oils adulterated with soybean oil, corn oil, or rapeseed oil demonstrated the effectiveness of our method. The proposed approach not only identifies unknown adulteration during practical inspections but also offers a novel method to address the representativeness issue of training set samples in model building. Furthermore, this method can be applied to other oils and food products, presenting a new perspective and solution for untargeted adulteration detection in market surveillance.

## Figures and Tables

**Table 1:** Fatty acid composition of avocado oils.

Fatty Acid ^a^ (%)	Production Area ^b^							Mean (n = 34)
	Mexico (n = 10)	New Zealand (n = 8)	France (n = 7)	Australia (m = 3)	USA (n = 2)	Spain (n = 2)	Kenya (n = 2)	
C16:0	10.15	13.89	13.46	15.36	16.71	12.90	11.44	12.80
C16:1	3.16	3.96	3.56	5.89	5.95	2.35	4.13	3.85
C17:0	0.03	0.05	0.05	0.04	0.05	0.07	0.04	0.05
C17:1	0.06	0.10	0.09	0.09	0.10	0.12	0.06	0.08
C18:0	2.10	1.38	1.76	0.50	1.15	2.43	1.98	1.67
C18:1	73.24	68.75	68.27	67.61	63.57	69.85	65.50	69.44
C18:2	10.36	10.78	11.75	9.71	11.46	11.08	15.75	11.11
C18:3	0.48	0.65	0.58	0.52	0.69	0.59	0.72	0.58
C20:0	0.21	0.22	0.26	0.10	0.16	0.36	0.20	0.22
C20:1	0.21	0.21	0.21	0.17	0.17	0.26	0.18	0.20
SFA	12.49	15.55	15.54	16.00	18.07	15.76	13.66	14.74
MUFA	76.67	73.02	72.13	73.77	69.79	72.57	69.87	73.57
PUFA	10.85	11.44	12.33	10.23	12.14	11.67	16.47	11.69
PUFA/SFA	0.91	0.74	0.81	0.65	0.69	0.74	1.31	0.83

^a^ SFA, saturated fatty acid; MUFA, monounsaturated fatty acid; PUFA, polyunsaturated fatty acid. ^b^ Relative content, %.

## Data Availability

The original contributions presented in the study are included in the article; further inquiries can be directed to the corresponding authors.
